# Eight novel hepatitis C virus genomes reveal the changing taxonomic structure of genotype 6

**DOI:** 10.1099/vir.0.047506-0

**Published:** 2013-01

**Authors:** Hongren Wang, Zhiguo Yuan, Eleanor Barnes, Manqiong Yuan, Chunhua Li, Yongshui Fu, Xueshan Xia, Gang Li, Paul N. Newton, Manivanh Vongsouvath, Paul Klenerman, Oliver G. Pybus, Donald Murphy, Kenji Abe, Ling Lu

**Affiliations:** 1 Vaccine Institute, 3rd Affiliated Hospital of Sun Yat-sen University, Guangzhou, Guangdong, PR China; 2 The Peter Medawar Building for Pathogen Research, University of Oxford, South Parks Road, OX1 3SY, UK; 3 The Viral Oncology Center, Department of Pathology and Laboratory Medicine, University of Kansas Medical Center, Kansas City, Kansas, USA; 4 Guangzhou Blood Center, Guangzhou, Guangdong, PR China; 5 Faculty of Life Science and Technology, Kunming University of Science and Technology, Kunming, Yunnan, PR China; 6 Wellcome Trust-Mahosot Hospital-Oxford Tropical Medicine Research Collaboration, Microbiology Laboratory, Mahosot Hospital, Vientiane, Lao PDR; 7 Centre for Clinical Vaccinology and Tropical Medicine, Churchill Hospital, Nuffield Department of Medicine, University of Oxford, Oxford OX3 7LJ, UK; 8 Department of Zoology, University of Oxford, South Parks Road, OX1 3PS, UK; 9 Institut National de Santé Publique du Québec, Laboratoire de Santé Publique du Québec, Sainte-Anne-de-Bellevue, QC, Canada; 10 Department of Pathology, National Institute of Infectious Diseases, Shinjuku-ku, Tokyo 162-8640, Japan

## Abstract

Analysis of partial hepatitis C virus sequences has revealed many novel genotype 6 variants that cannot be unambiguously classified, which obscure the distinctiveness of pre-existing subtypes. To explore this uncertainty, we obtained genomes of 98.0–98.8 % full-length for eight such variants (KM35, QC273, TV257, TV476, TV533, L349, QC271 and DH027) and characterized them using phylogenetic analyses and per cent nucleotide similarities. The former four are closely related phylogenetically to subtype 6k, TV533 and L349 to subtype 6l, QC271 to subtypes 6i and 6j, and DH027 to subtypes 6m and 6n. The former six defined a high-level grouping that comprised subtypes 6k and 6l, plus related strains. The threshold between intra- and inter-subtype diversity in this group was indistinct. We propose that similar results would be seen elsewhere if more intermediate variants like QC271 and DH027 were sampled.

The hepatitis C virus (HCV) is genetically highly variable and is currently classified into six confirmed and one provisional genotype. Among them, genotype 6 exhibits the greatest genetic diversity and has been proposed to have an older evolutionary origin than other HCV genotypes (Salemi & Vandamme, 2002). Divergent isolates of genotype 6 have been found exclusively in South-east Asia or among emigrants from there, suggesting that the strains are endemic to that region (Bernier *et al.*, 1996; Mellor *et al.*, 1996; Noppornpanth *et al.*, 2006; Shinji *et al.*, 2004; Stuyver *et al.*, 1995; Simmonds *et al.*, 1996; Thaikruea *et al.*, 2004; Theamboonlers *et al.*, 2002). Taxonomically, as many as 23 subtypes of genotype 6 (6a–6w) have been assigned and for each at least one full-length genome sequence has been characterized (Kuiken *et al.*, 2005). Whole genome sequences are the gold standard for genetic and evolutionary analysis of HCV and for accurate classification. Measuring the extent of HCV diversity is essential not only for understanding the origin and evolution of HCV, but also for defining new preventive strategies and developing novel therapies and vaccines.

The current HCV nomenclature confirms the designation of genotypes and subtypes based on phylogenetic analysis of full-length genome sequences. In terms of nucleotide identity a difference of 31–33 % is required to discriminate genotypes, while for subtypes no such fixed criterion is proposed because they are thought to represent an epidemiological phenomenon associated with their recent spreads. However, all the currently designated subtypes do show nucleotide differences by >15 % (Simmonds *et al.*, 2005). Using partial genome sequences we have previously found a number of novel HCV-6 variants whose nucleotide distances from the currently defined subtypes are around 15 %, making their classification ambiguous. This ambiguity is reflected in phylogenetic analyses: some subtypes are distinct and separated by long internal branches, whereas other subtypes are more closely related and sometimes seem to merge into a single but larger phylogenetic group. Here, we demonstrate this by generating and analysing 98.0–98.8 % of full-length genome sequences from six variants related to subtypes 6k and 6l (KM35, QC273, TV257, TV476, TV533 and L349). In addition, we also determined such sequences for two other HCV-6 variants (DH027 and QC271) that appear to not fall within any currently known subtypes.

HCV genomes were determined each with 22–30 overlapping amplicons for the following 10 strains: KM35, QC273, TV257, TV476, TV533, L349, TV317, TV494, D027 and QC271. Their lengths ranged from 9412 to 9533 nt, corresponding to the nucleotide numbering of 1 to 9452–9564 in the H77 genome, covering 98.0–98.8 % of the full-length. The 5′ UTRs were all 338 nt long, while the 3′ UTRs varied from 23 to 144 nt long. Six isolates (KM35, QC273, TV476, TV533, D027 and QC271) had their 3′ UTRs amplified through to the poly(U) tract, but for four isolates (TV257, L349, TV317 and TV494) the poly(U) tracts were not obtained. Isolates KM35, QC273 and TV317 each contain a single ORF of 9048 nt. TV257, TV476, TV533, L349, TV494 and QC271 each contain an ORF of 9051 nt, while the ORF of DH027 is 9054 nt long. The sizes of the 10 HCV protein encoding regions were as follows: core (573 nt/191 aa), E1 (576 nt/192 aa), E2 (1092–1098 nt/364–366 aa), P7 (189 nt/63 aa), NS2 (651 nt/217 aa), NS3 (1893 nt/631 aa), NS4A (162 nt/54 aa), NS4B (783 nt/261 aa), NS5A (1350–1353 nt/450–451 aa) and NS5B (1776 nt/591 aa) (see Table S1, available in JGV Online).

TV317 and TV494 grouped closely with two isolates of subtype 6l: D33 and 537796 (Fig. 1). Since this grouping is unambiguous, the classification of TV317 and TV494 will no longer be discussed. Each of the remaining eight variants was pairwise compared with the 54 reference sequences shown in Fig. 1(a). These reference strains represent the 23 subtypes (6a–6w) currently assigned under genotype 6. They included five genomes of subtype 6a, four genomes each of subtypes 6e, 6m, 6n and 6t, three genomes each of subtypes 6f, 6i, 6o, 6u, 6v and 6w, two genomes each of subtypes 6g, 6j and 6l, and one representative each from subtypes 6b, 6c, 6d, 6h, 6k, 6p, 6q, 6r and 6s. When compared to each other, the eight novel variants showed nucleotide similarities of 76.7–83.7 % across the whole genome and of 76.0–83.2 % across the entire ORF (Table S2). When compared to the 54 reference sequences, their nucleotide similarities were 72.2–86.2 % across the whole genome and 71.4–85.7 % across the entire ORF (Table S3). Within the 10 viral genes, core and NS5B showed the highest similarities, whilst P7 and NS2 the lowest (Table S4).

**Fig. 1.**
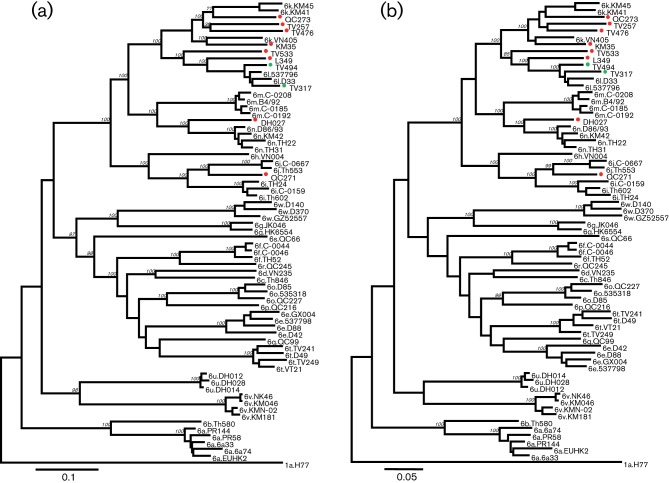
Phylogenetic trees estimated from (a) complete nucleotide sequences and (b) predicted amino acid sequences. Reference HCV sequences are each indicated by a subtype name followed by an isolate name. KM35, QC273, TV257, TV476, TV533, L349, D027 and QC271 represent the eight novel genotype 6 variants completely sequenced in this study and are indicated each with a red circle. TV317 and TV494 are two 6l isolates that were also completely sequenced in this study; they were marked each with a green circle. Bootstrap analysis values of ≥70 % are shown in italics. Bars indicate a genetic distance of 0.10 nucleotide or 0.05 amino acid substitutions per site.

Of the eight novel variants, six (KM35, QC273, TV257, TV476, TV533 and L349) were found to be roughly equally similar to subtypes 6k and 6l. The former four (KM35, QC273, TV257 and TV476) are found to be more closely related, but remaining somewhat distant, to 6k (isolate VN405) than to 6l. These four exhibit nucleotide similarities of 83.2–85.8 % to 6k, and of 80.7–81.4 % to 6l. Conversely, isolates TV533 and L349 exhibit nucleotide similarities of 82.7–86.2 % to 6l, and of 80.5–81.0 % to 6k. Recently, we have characterized two variants KM41 and KM45 that are related to 6k (Lu *et al.*, 2006) and exhibit nucleotide similarities of 83.3–83.4 % to VN405, which is the prototype isolate of 6k. Likewise, QC271 was roughly equally similar to subtypes 6i and 6j, whilst DH027 was roughly equally similar to subtypes 6m and 6n. QC271 exhibits nucleotide similarities of 85.2–85.5 % to 6j and of 83.0–83.8 % to 6i, whilst DH027 displays nucleotide similarities of 83.9–85.0 % to 6n and of 81.0–81.3 % to 6m. The nucleotide similarities of the genomes described above fall close to the threshold by which different subtypes of HCV are discriminated making their classification difficult.

A phylogenetic tree was estimated using the obtained genome sequences. The phylogeny showed that isolates KM35, QC273, TV257 and TV476 formed a loose cluster with VN405, KM41 and KM45. Within this cluster, three subsets can be divided. The first contains KM41, KM45 and QC273, the second contains TV257 and TV456, and the third contains KM35 and VN405. Genetic distances among the three subsets (18.2–18.6 %) are comparable to those between subtypes 6f and 6r (19.3–19.8 %), 6i and 6j (18.5–19.4 %) and 6m and 6n (20.8–22.9 %). Isolates TV533 and L349 were loosely grouped in a second cluster with four 6l isolates (537796, D33, L349 and TV494). Taken together, these two clusters form a larger group that contains 13 isolates related to subtypes 6k and 6l. The internal branch lengths that separate lineages in this group appear smaller than in the remainder of the HCV genotype 6 tree (Fig. 1a).

In addition to subtypes 6k and 6l, there are other well-supported taxonomic groupings above the subtype level: subtypes 6m and 6n cluster strongly together, as also do subtypes 6h, 6i and 6j. The isolate DH027 was placed between 6m and 6n, whilst isolate QC271 was placed between 6i and 6j. The addition of DH027 and QC271 clearly interrupts the separation of 6m/6n and 6i/6j (Lu *et al.*, 2007). There was strong bootstrap support for a group comprising subtypes 6k, 6l, 6m, 6n, 6h, 6j, 6i and their related viruses, and all the eight novel variants reported here belong to this clade. We estimated a second phylogeny using predicted amino acid sequences (Fig. 1b) and its topology was consistent with the nucleotide phylogeny in Fig. 1(a). Sequences from the ten protein-coding regions were also analysed separately, and similar structures were obtained (data not shown).

It is possible that the phylogenetic tree shape may be affected by recent viral recombination events that occurred between subtypes 6k and 6l, between 6i and 6j, and between 6m and 6n. To investigate this, pairwise similarity scores were calculated between the eight novel variants and the 54 reference sequences that represent subtypes 6a–6w by using the rdp software. In each case, similar plot patterns were observed but no evidence of recent viral recombination events was seen (data not shown).

In this study, HCV genomes of 98.0–98.8 % full-length were determined for eight novel genotype 6 variants (DH027, KM35, L349, QC271, QC273, TV257, TV476 and TV533). All those except for DH027 and QC271 were classified into a large cluster containing both subtypes 6k and 6l. Of them, six were each distant from the prototypic isolates of 6k and 6l. Within this cluster there are several short internal branches above the subtype level; such branches are rare in the rest of the genotype 6 phylogeny, and represent active viral transmission in the distant past. One explanation is that the 6k/6l-related group has been sampled more densely, such that the long internal branches present in other parts of the tree represent insufficient sampling: the phylogenetic positions of DH027 and QC271 (which are both equidistant between pairs of subtypes) further support this notion. Other pairs of subtypes that appear to be clearly separated (e.g. 6a/6b, 6c/6d, 6 g/6w, 6o/6p, 6q/6t, 6u/6v etc.) may therefore become interrupted and less distinct as further diversity is uncovered. This is likely to be the case once further molecular epidemiology studies of HCV are completed in South-east Asian countries in which there is currently a lack of extensive HCV surveillance. It is interesting to note that a breakdown in subtype distinctiveness has also been described for human immunodeficiency virus type 1 (HIV-1): widespread surveillance and sampling of HIV-1 from central Africa (Vidal *et al.*, 2000) largely eroded the long internal branches that previously had defined highly distinct HIV-1 subtypes (Rambaut *et al.*, 2001).

Analysis of our eight novel variants revealed two features: (i) they are slightly more distinct from subtype prototype sequence than other strains, making their subtype assignment more difficult; (ii) a larger cluster comprising subtypes 6k, 6l and related viruses exists, representing a more ancient phylogenetic grouping. A similar grouping of 6i/6j and 6m/6n could be defined if more variants like DH027 and QC271 are found. Further groupings of subtypes, specifically 6f/6r and 6a/6b, are strongly suggested by the existence of isolates that appear to be placed between the subtypes in each pair (data not shown); these isolates have yet to be entirely sequenced. We therefore hypothesize that many HCV variants are still unsampled and represent an important missing component of global HCV diversity, within which there may be less or no clear separation of subtypes. If this is the case then there could be an unmanageable profusion of subtype designations in the future.

A total of 10 serum samples was used in this study. KM35 was from a voluntary blood donor and DH027 was from an HIV-1-infected injection drug user; both were originally from Kunming City, Yunnan Province, China (Fu *et al.*, 2011; Xia *et al.*, 2008). Isolates TV257, TV317, TV476, TV494 and TV533 were all from blood donors from Ho Chi Minh City, Vietnam (Pham *et al.*, 2011). L349 was from a patient in Vientiane city, Lao PDR (Laos) (Syhavong *et al.*, 2010; Pybus *et al.*, 2009). QC271 and QC273 were sampled in Quebec, Canada from individuals who had the origins from Thailand and Cambodia, respectively (Murphy *et al.*, 2007). These samples were selected because our preliminary analyses of their partial core–E1 sequences have shown ambiguous classification between subtypes.

The genome sequence of each HCV isolate was determined from 100 µl of serum using the methods described previously (Li *et al.*, 2006). In brief, RNA was extracted using Tripure (Roche). cDNA was transcribed using AMV reverse transcriptase (Roche) and random hexamers (Promega). Overlapping fragments were amplified using the Fast Start PCR system (Roche) with the primers listed in Table S5. To avoid PCR false positives, standard procedures were taken (Kwok & Higuchi, 1989). At least one negative control, one positive control and a water blank were included in each of the following steps: RNA extraction, reverse transcription and the 1st and 2nd rounds of PCR. After PCR, the amplicons were purified using QIAquick PCR purification kit (Qiagen) according to the manufacturer’s protocol. To obtain consensus sequences to reflect the heterogeneity of viral population within each individual, the purified amplicons were sequenced directly. The sequencing was done in both directions by using ABI Prism BigDye 3.0 terminators with an appropriate primer on an ABI Prism 3500 genetic analyser (PE Applied Biosystems). The resulting chromatograms were corrected using SeqMan in the dnastar package (dnastar Inc.). The finalized sequences were aligned using BioEdit (Tippmann, 2004) followed by manual adjustments and corrections.

Maximum-likelihood phylogenetic trees were estimated using PHYML (Guindon & Gascuel, 2003) under the GTR+I+Γ_6_ nucleotide substitution model. The transition/transversion rate ratio, the proportion of invariable sites, and the gamma distribution shape parameter were estimated from the alignment. Base frequencies were adjusted to maximize the likelihood. Bootstrap resampling was performed in 500 replicates. For pairwise sequence comparisons, nucleotide similarities were calculated using mega5 (Kumar *et al.*, 2004) and genetic distances displayed from the tree file.

To detect possible virus recombination events, we used rdp3 (Recombination Detection Program, version 3) (Martin *et al.*, 2010). The program was run under default settings with the following adjustments: (i) window size was set to 40 nt; (ii) linear sequences option was chosen; (iii) six different methods (rdp, geneconv, MaxChi, Bootscan, Chimaera and SiScan) were performed simultaneously on the multiple sequence alignment; and (iv) only events detected by more than two methods were listed.

## References

[r1] Bernier L. Willems B. Delage G. Murphy D. G. **(**1996**).** Identification of numerous hepatitis C virus genotypes in Montreal, Canada. J Clin Microbiol 34, 2815–2818 8897188 10.1128/jcm.34.11.2815-2818.1996 PMC229409

[r2] Fu Y. Wang Y. Xia W. Pybus O. G. Qin W. Lu L. Nelson K. **(**2011**).** New trends of HCV infection in China revealed by genetic analysis of viral sequences determined from first-time volunteer blood donors. J Viral Hepat 18, 42–52 10.1111/j.1365-2893.2010.01280.x 20196805 PMC3020328

[r3] Guindon S. Gascuel O. **(**2003**).** A simple, fast, and accurate algorithm to estimate large phylogenies by maximum likelihood. Syst Biol 52, 696–704 10.1080/10635150390235520 14530136

[r4] Kuiken C. Yusim K. Boykin L. Richardson R. **(**2005**).** The Los Alamos hepatitis C sequence database. Bioinformatics 21, 379–384 10.1093/bioinformatics/bth485 15377502

[r5] Kumar S. Tamura K. Nei M. **(**2004**).** mega3: Integrated software for Molecular Evolutionary Genetics Analysis and sequence alignment. Brief Bioinform 5, 150–163 10.1093/bib/5.2.150 15260895

[r6] Kwok S. Higuchi R. **(**1989**).** Avoiding false positives with PCR. Nature 339, 237–238 10.1038/339237a0 2716852

[r7] Li C. Fu Y. Lu L. Ji W. Yu J. Hagedorn C. H. Zhang L. **(**2006**).** Complete genomic sequences for hepatitis C virus subtypes 6e and 6g isolated from Chinese patients with injection drug use and HIV-1 co-infection. J Med Virol 78, 1061–1069 10.1002/jmv.20663 16789024

[r8] Lu L. Nakano T. Li C. Fu Y. Miller S. Kuiken C. Robertson B. H. Hagedorn C. H. **(**2006**).** Hepatitis C virus complete genome sequences identified from China representing subtypes 6k and 6n and a novel, as yet unassigned subtype within genotype 6. J Gen Virol 87, 629–634 10.1099/vir.0.81400-0 16476984

[r9] Lu L. Li C. Fu Y. Thaikruea L. Thongswat S. Maneekarn N. Apichartpiyakul C. Hotta H. Okamoto H. **& other authors (**2007**).** Complete genomes for hepatitis C virus subtypes 6f, 6i, 6j and 6m: viral genetic diversity among Thai blood donors and infected spouses. J Gen Virol 88, 1505–1518 10.1099/vir.0.82604-0 17412980

[r10] Martin D. P. Lemey P. Lott M. Moulton V. Posada D. Lefeuvre P. **(**2010**).** rdp3: a flexible and fast computer program for analyzing recombination. Bioinformatics 26, 2462–2463 10.1093/bioinformatics/btq467 20798170 PMC2944210

[r11] Mellor J. Walsh E. A. Prescott L. E. Jarvis L. M. Davidson F. Yap P. L. Simmonds P. **(**1996**).** Survey of type 6 group variants of hepatitis C virus in Southeast Asia by using a core-based genotyping assay. J Clin Microbiol 34, 417–423 8789027 10.1128/jcm.34.2.417-423.1996 PMC228809

[r12] Murphy D. G. Willems B. Deschênes M. Hilzenrat N. Mousseau R. Sabbah S. **(**2007**).** Use of sequence analysis of the NS5B region for routine genotyping of hepatitis C virus with reference to C/E1 and 5′ untranslated region sequences. J Clin Microbiol 45, 1102–1112 10.1128/JCM.02366-06 17287328 PMC1865836

[r13] Noppornpanth S. Sablon E. De Nys K. Lien T. X. Brouwer J. Van Brussel M. Smits S. L. Poovorawan Y. Osterhaus A. D. M. E. Haagmans B. L. **(**2006**).** Genotyping hepatitis C viruses from Southeast Asia by a novel line probe assay that simultaneously detects core and 5′ untranslated regions. J Clin Microbiol 44, 3969–3974 10.1128/JCM.01122-06 16957039 PMC1698374

[r14] Pham V. H. Nguyen H. D. Ho P. T. Banh D. V. Pham H. L. Pham P. H. Lu L. Abe K. **(**2011**).** Very high prevalence of hepatitis C virus genotype 6 variants in southern Vietnam: large-scale survey based on sequence determination. Jpn J Infect Dis 64, 537–539 22116339 PMC3743674

[r15] Pybus O. G. Barnes E. Taggart R. Lemey P. Markov P. V. Rasachak B. Syhavong B. Phetsouvanah R. Sheridan I. **& other authors (**2009**).** Genetic history of hepatitis C virus in East Asia. J Virol 83, 1071–1082 10.1128/JVI.01501-08 18971279 PMC2612398

[r16] Rambaut A. Robertson D. L. Pybus O. G. Peeters M. Holmes E. C. **(**2001**).** Human immunodeficiency virus. Phylogeny and the origin of HIV-1. Nature 410, 1047–1048 10.1038/35074179 11323659

[r17] Salemi M. Vandamme A. M. **(**2002**).** Hepatitis C virus evolutionary patterns studied through analysis of full-genome sequences. J Mol Evol 54, 62–70 10.1007/s00239-001-0018-9 11734899

[r18] Shinji T. Kyaw Y. Y. Gokan K. Tanaka Y. Ochi K. Kusano N. Mizushima T. Fujioka S. Shiraha H. **& other authors (**2004**).** Analysis of HCV genotypes from blood donors shows three new HCV type 6 subgroups exist in Myanmar. Acta Med Okayama 58, 135–142 15471435 10.18926/AMO/32110

[r19] Simmonds P. Mellor J. Sakuldamrongpanich T. Nuchaprayoon C. Tanprasert S. Holmes E. C. Smith D. B. **(**1996**).** Evolutionary analysis of variants of hepatitis C virus found in South-East Asia: comparison with classifications based upon sequence similarity. J Gen Virol 77, 3013–3024 10.1099/0022-1317-77-12-3013 9000092

[r20] Simmonds P. Bukh J. Combet C. Deléage G. Enomoto N. Feinstone S. Halfon P. Inchauspé G. Kuiken C. **& other authors (**2005**).** Consensus proposals for a unified system of nomenclature of hepatitis C virus genotypes. Hepatology 42, 962–973 10.1002/hep.20819 16149085

[r21] Stuyver L. Wyseur A. van Arnhem W. Lunel F. Laurent-Puig P. Pawlotsky J. M. Kleter B. Bassit L. Nkengasong J. **& other authors (**1995**).** Hepatitis C virus genotyping by means of 5′-UR/core line probe assays and molecular analysis of untypeable samples. Virus Res 38, 137–157 10.1016/0168-1702(95)00052-R 8578855

[r22] Syhavong B. Rasachack B. Smythe L. Rolain J. M. Roque-Afonso A. M. Jenjaroen K. Soukkhaserm V. Phongmany S. Phetsouvanh R. **& other authors (**2010**).** The infective causes of hepatitis and jaundice amongst hospitalised patients in Vientiane, Laos. Trans R Soc Trop Med Hyg 104, 475–483 10.1016/j.trstmh.2010.03.002 20378138 PMC2896487

[r23] Thaikruea L. Thongsawat S. Maneekarn N. Netski D. Thomas D. L. Nelson K. E. **(**2004**).** Risk factors for hepatitis C virus infection among blood donors in northern Thailand. Transfusion 44, 1433–1440 10.1111/j.1537-2995.2004.04073.x 15383015

[r24] Theamboonlers A. Chinchai T. Bedi K. Jantarasamee P. Sripontong M. Poovorawan Y. **(**2002**).** Molecular characterization of hepatitis C virus (HCV) core region in HCV-infected Thai blood donors. Acta Virol 46, 169–173 12580379

[r25] Tippmann H. F. **(**2004**).** Analysis for free: comparing programs for sequence analysis. Brief Bioinform 5, 82–87 10.1093/bib/5.1.82 15153308

[r26] Vidal N. Peeters M. Mulanga-Kabeya C. Nzilambi N. Robertson D. Ilunga W. Sema H. Tshimanga K. Bongo B. Delaporte E. **(**2000**).** Unprecedented degree of human immunodeficiency virus type 1 (HIV-1) group M genetic diversity in the Democratic Republic of Congo suggests that the HIV-1 pandemic originated in Central Africa. J Virol 74, 10498–10507 10.1128/JVI.74.22.10498-10507.2000 11044094 PMC110924

[r27] Xia X. Lu L. Tee K. K. Zhao W. Wu J. Yu J. Li X. Lin Y. Mukhtar M. M. **& other authors (**2008**).** The unique HCV genotype distribution and the discovery of a novel subtype 6u among IDUs co-infected with HIV-1 in Yunnan, China. J Med Virol 80, 1142–1152 10.1002/jmv.21204 18461611 PMC2999914

